# Clozapine for Patients With Intellectual Disabilities: A Case Series Illustrating the Clinical Potentials

**DOI:** 10.1155/crps/9592396

**Published:** 2025-10-28

**Authors:** Magnus Roland Balleby, Jacob Bentsen, Jimmi Nielsen

**Affiliations:** Mental Health Center Glostrup, Copenhagen University Hospital, Glostrup, Denmark

**Keywords:** aggressive behaviour, clozapine, intellectual disability, persistent challenging behaviour, point-of-care test, self-injurious behaviour

## Abstract

**Background:**

Challenging behaviours (CBs) are common in patients with intellectual disabilities (IDs) and diagnosing an underlying primary psychiatric disorder is often difficult. Even though many respond to non-pharmacological or pharmacological interventions persistent aggressive and self-injurious behaviour occur and no treatment-resistant guideline exists. We aim to present clinical scenarios regarding patients with ID and persistent CB with or without a primary psychiatric disorder where clozapine could be considered.

**Case Presentation:**

We present five patients with ID with persistent CB with or without a primary psychiatric disorder treated with clozapine. Four of the five patients responded well to clozapine treatment with markedly decreased CB, and two patients had reduced psychotic- or affective symptoms. Side-effects were mild and manageable. Haematological monitoring was performed with a point-of-care (POC) test device.

**Conclusions:**

We show that clozapine can be efficacious in persistent CB and/or treatment-resistant psychiatric symptoms in patients with ID. Monitoring and managing side-effects were possible.

**Impact and Implications:**

We suggest that clozapine should be considered in patients with ID regardless of a primary psychiatric disorder when CB does not respond to non-pharmacological and first line pharmacological treatment. It is possible to monitor and manage side-effects with a systematic approach including the use of POC testing.

## 1. Introduction

Clozapine remains the golden standard for treatment-resistant schizophrenia (TRS) and suicide prevention in patients with schizophrenia. In addition, clozapine has demonstrated efficacy in treatment-resistant bipolar disorder [1] and borderline personality [2]. Clozapine is also well-recognised for its anti-aggressive effect, that seems to be independent of sedation and its antipsychotic effect in patients with schizophrenia [3]. A similar anti-aggressive effect has been demonstrated in schizophrenia patients with executive dysfunction [4], autism [5] and dementia [6].

Intellectual disability (ID) is characterised by reduced mental functioning and adaptive abilities. People with ID ranges from less impairment to people who require high level of support. Mental disorders in patients with mild IDs are comparable to the disorders seen in the people without ID. In contrast, patients with severe IDs might not be able to describe symptoms that give rise to a mental disorder, such as schizophrenia, due to lack of ability to express themselves. At the same time, mental disorders are more likely to present as challenging behaviour (CB), such as aggressive and self-injurious behaviour. However, the aetiology of CB is diverse, and besides psychopathology, it also includes medical, social and psychological problems [7]. CB are more common in patients with severe IDs [8], and is roughly present in one out of five people with ID [8, 9].

Currently, no treatment guidelines exist specifically for patients with ID and mental disorder, such as schizophrenia and bipolar disorder, therefore treatment should follow standard guidelines.

In case of no clearly identified mental disorder, CB in patients with IDs should mainly be managed with non-pharmacological interventions, such as social support and cognitive behavioural therapy. If the response is insufficient, antipsychotic drugs, typically aripiprazole or risperidone, are often used [10]. Although a recent meta-analysis has confirmed the effect of antipsychotics on CB, persistent CB may still occur [11].

In TRS, initiation of clozapine treatment is delayed compared to the recommendations in treatment guidelines [12]. Managing safety and tolerability of clozapine in ID patients remains an additional challenge, because ID patients often lack the language to convey side effects and have difficulties participating in the haematological monitoring during clozapine treatment. However, a previous Danish naturalistic study found reduced hospitalisation in ID patients with and without a mental disorder [13], suggesting that treatment with clozapine may be beneficial in this patient group.

With this case series, we aim to present clinical scenarios where clozapine could be considered regardless of whether a mental disorder is present. We included five patients with ID and persistent CB and/or psychotic and affective symptoms.

## 2. Procedure and Case Presentation

All patients were recruited from the Unit for IDs at Mental Health Centre Glostrup, Capital Region of Denmark; a tertiary specialised outpatient clinic with catchment area of 1.9 million inhabitants. The primary inclusion criteria were adult with ID and a comorbid mental disorder or CB. In mild ID the diagnosis was confirmed by using the Wechsler Adult Intelligence Scale (WAIS) [14], for severe ID the diagnosis was confirmed by two experienced psychiatric consultants according to ICD-10 criteria [15].

We identified all patients treated with clozapine and reviewed their treatment courses. Out of these, five cases were purposely selected by the authors to illustrate the diversity of clinical scenarios where clozapine has been used.

According to Danish legislation, written consent were obtained from the patients' legal guardians because the five patients did not have the competence to give informed consent.

We chose the label patient with ID instead of people with ID because we are referring to a specific group in contact with mental health service.

### 2.1. Measures

Patients were retrospectively rated with Clinical Global Impresssion-Severity of illness (CGI-S) [16] at time of initiation of clozapine and at the time of preparation of the manuscript. Consensus scores were obtained with the psychiatrists and other clinical staff in the outpatient clinic.

Side-effects were systematically screened minimum every 3 months with the rating scale Udvalg for Kliniske Undersøgelser (UKU) [17] by experienced nurses and doctors in collaboration with the patient, residential staff, and the patient's family.

The specialised clinic had 350 patients with ID and out of those 22 patients (6.3%) were treated with clozapine. An overview of the five patients can be seen in Table 1 including dosing of clozapine, plasma levels of clozapine and side effects.

### 2.2. Case Presentations

#### 2.2.1. Case A

A 53-year-old woman with a history of encephalitis at the age of 6 months as the likely cause of profound ID. She had previously been involuntarily hospitalised due to violent episodes and had required mechanical restraint following assaults on the hospital staff.

Before initiating clozapine, the patient was treated with zuclopenthixol up to 130 mg per day in order to reduce aggressive behaviour. The patient was restless, self-harming, aggressive, appeared emotionally distressed and was losing weight. Previously blood sampling was only feasible with general anaesthesia but the introduction of a point-of-care (POC) test [18] for haematological monitoring made clozapine treatment an option. Two weeks after initiation of clozapine, the patient was calmer and less agitated.

Over a 6-month period, zuclopenthixol was reduced and discontinued, while the dose of clozapine was fixed. Two months after the discontinuation of zuclopenthixol, the patient became more restless and aggressive towards staff. Clozapine dose was increased and zuclopenthixol was reinstated as pro re nata (PRN). The following month, the aggressive behaviour decreased.

At the latest checkup at our clinic, 2 years and 9 months after the initiation of clozapine, the patient's aggressive episodes were significantly reduced and the patient appeared less appeared emotionally distressed.

#### 2.2.2. Case B

A 47-year-old man with childhood autism and severe ID of unknown cause. The patient experienced shorter periods of psychotic/manic-like symptoms, with suspected visual hallucinations, signs of thought disorders and with restlessness and aggressive behaviour. He had previously been involuntarily hospitalised due to aggressiveness. The patient also engaged in self-injurious behaviour, such as biting himself.

Before the initiation of clozapine, the patient was treated with olanzapine, zuclopenthixol, alprazolam and lamotrigine. The behaviour was threatening, unpredictable and violent toward staff and co-residents.

One month after the initiation of clozapine, the staff noticed a slight improvement in aggressive behaviour and the patient was calmer, making it easier to correct his behaviour. In the following 3 months, there were no incidents of serious aggressive outbursts.

At the latest checkup, nearly 2 years after the initiation of clozapine, the patient's aggressive behaviour remained reduced, and his overall well-being had improved. Slowly tapering of concomitant medications besides clozapine are still in progress.

#### 2.2.3. Case C

A 68-year-old woman with atypical autism, moderate ID of unknown cause, encephalopathy and essential hypertension.

The patient had episodes of aggressive behaviour, both verbally and physically, against caregivers and co-residents. She had been hospitalised several times in the past for treatment of depression with mixed features and psychosis. She had been briefly admitted to the neurological department because of a generalised tonic-clonic seizure.

Prior to the initiation of clozapine, the patient was treated with lamotrigine, olanzapine and oxazepam. Her condition was fluctuating, and she exhibited self-injurious behaviour, e.g., scratching her face. She was excited and cried at the same time with sleep disruption which was interpreted as a mixed affective episode.

After 2 weeks of clozapine treatment, the patient's overall condition had improved. She was calmer, less engaged in self-injurious behaviour and her sleep had improved. However, a week later, the staff reported that the previous CB had returned.

Five months later, the CB continued, and the dose of clozapine was increased and 3 months later, the patient was observed to be more settled, and her anger outbursts had decreased. However, nearly 2 years after the initiation of clozapine, it became evident that the condition remained fluctuating, and the overall effect of clozapine was questioned. Consequently, it was decided that clozapine should be tapered off. Currently, the clozapine dose has been reduced from 250 to 150 mg with no clear signs of worsening.

#### 2.2.4. Case D

A 31-year-old man with mild ID of unknown cause, pervasive developmental disorder and persistent delusional disorder. His most prominent psychotic symptom was auditory hallucinations in which he heard the voice of the devil.

Before the initiation of clozapine, he had been admitted to psychiatric wards multiple times and on two occasions mechanical restrained due to aggressive behaviour. He had also been brought to the psychiatric emergency department by the police several times because of aggressive behaviour.

Clozapine was initiated and zuclopenthixol was slowly tapered off. After 2 weeks, the patient appeared less emotionally distressed, and his overall well-being had improved.

Over the next 29 months, the patient's overall well-being had improved, and the auditory hallucinations had subsided. It was noticed that the patient was tyred, and therefore the dose of clozapine was reduced. Within 10 days after the dose reduction of clozapine, the patient once again was experiencing auditory hallucinations. The dose of clozapine was increased, and in the last two and half years (5 years after the initiation of clozapine), the auditory hallucinations had subsided, and the CB had decreased markedly.

#### 2.2.5. Case E

A 56-year-old man with autism, profound ID of unknown cause and impaired vision. The patient had a long-term history of aggressive- and self-injurious behaviour. He had assaulted caregivers and co-residents several times.

Before the initiation of clozapine, the patient was treated with valproate, zuclopenthixol and clonazepam (PRN). Two weeks after the initiation of clozapine, the patient's overall well-being had improved. He was happier, calmer and his sleep had improved. He was less aggressive and less prone to self-injurious behaviour. The staff could more easily guide the patient, and his ability to delay gratification based on need had increased.

One year later, the patient remained less aggressive with no self-injurious behaviour. There was a minor short-term destabilisation, likely due to a reduction in the dose of zuclopenthixol. Ten months later the overall well-being had further improved, with no outwardly aggressive behaviour and significantly reduced self-injurious behaviour. Three years after initiating of clozapine the situation remained unchanged. The improved behaviour made it possible that he could remain at his current place of residence.

## 3. Discussion

The main findings of this case series are that clozapine may be an efficacious and safe antipsychotic drug to be used in patients with ID in a variety of clinical scenarios, where diagnosing of mental disorder is challenging. The clinical scenarios included patients with aggressive or self-injurious behaviour in patients with or without a mental disorder. The severity of the ID ranged from mild to profound (IQ <69 to IQ<20), and all the patients had autism spectrum disorder; both complicating the identification of psychotic symptoms.

Regardless of the underlying condition, four out of the five patients experienced a significant reduction in CB and increased overall well-being. The most prominent effect was seen on aggressive and self-injurious behaviour, which is in line with a newly published systematic review which found remission or a reduced frequency of aggressive and self-injurious behaviour in 85.7% of patients (*n* = 21) with ID [19]. Another measure of the effect of clozapine is the use of hospitalisations; none of the patients were hospitalised after the initiation of clozapine which is in line with findings from a Danish register study [13].

Clozapine successfully treated the psychotic symptoms of Case D. In the other cases psychotic symptoms could not be confirmed, but some of the CBs seen may have had a psychotic origin, and therefore the improvement could be due the antipsychotic effect of clozapine [20]. However, the non-specific effects of clozapine, mediated by histaminergic and noradrenergic antagonism, should not be neglected.

The underutilisation of clozapine on its main indication, TRS, is well-documented [21, 22]. Although no similar treatment algorithms exist for patients with ID, all the included cases had been treated with several psychotropics. The number of previous antipsychotics were four to eight for the patients with no confirmed psychotic symptoms (Case A + B + C + E) suggesting that all patients were persistent in terms of CB, affective- or psychotic symptoms. This could also suggest a similar hesitation of using clozapine in patients with ID, as seen in TRS. Supporting this further is the fact that several of the patients had been treated with other psychotropics before clozapine, such as antiepileptics, antihistamines, benzodiazepines and electroconvulsive therapy (ECT).

Case D differed from the rest of the cases, as this case only had mild ID and consequently the only patient able to describe psychotic symptoms. This may also explain why clozapine was initiated according to treatment algorithms [23], that is after not responding sufficiently to two antipsychotics.

The mandatory venous blood sampling is a well-known barrier for clozapine. Especially, patients with ID may not be able to collaborate on this procedure. In our case series, this was overcome by using a POC, measuring white blood corpuscles (WBCs) and absolute neutrophil count (ANC) with one drop of capillary blood. This approach has previously been used in patients with schizophrenia [24].

Although not supported by evidence, it is a general assumption that patients with ID are more prone to develop adverse effects to antipsychotics [25], which may add to the hesitance of using clozapine in this patient population. Side effects were mild constipation, weight gain, fatigue and hypersalivation, and thus comparable to previous findings in ID population [19] and in TRS in general [26]. The constipation was managed with over-the-counter laxative. Case D gained 21 kg over a period of 5 years and was started on semaglutide which led to a 5 kg weight loss. Case C had previously had a grand mal seizure before the initiation of clozapine, but there were no reported seizures on clozapine for Case C or the other patients.

This study should be interpreted within its limitations. The cases were purposely selected to illustrate the diversity of clozapine prescribing in our clinic. Although, we think that these patients are representative for our clinical experience with clozapine, we acknowledge the risk of selection bias.

We did not prospectively use formal rating scales, such as Positive and Negative Syndrome Scale [27] or the Overt Aggression Scale [28], to monitor CB, affective or psychotic symptoms. However, we retrospectively rated the patient on the CGI-S in consensus with involved staff. A method used in other case reports [29]. We cannot rule out that changes in the patients' environment contributed to the improvement in CB; however, none of the residential staff reported having made any substantial changes in their approach to the patients. Another limiting factor is that all patients were treated with several psychotropics, which may complicate determining the effect of clozapine. However, after introducing clozapine, no other psychotropics were initiated and clozapine facilitated a reduction in other psychotropics, which is in line with previous findings in TRS [30]. Finally, the fact that these patients were treated with polypharmacy represented real-life conditions in clinical practice.

The strengths of this case series included a long follow-up period of 2–5 years and high compliance with the clozapine treatment, as all medications were administered by the staff at the residential facilities. In addition, clozapine plasma levels were within the reference range. Finally, side effects were systematically screened minimum every 3 months with UKU [17].

## 4. Conclusion

These five cases illustrate that clozapine can be an effective treatment for various indications in patients with mild to profound ID. Managing the risk of adverse events is feasible with a systematic approach including the use of a POC test.

## Figures and Tables

**Table 1:** Overview of included patients treated with clozapine.

Sex Age	Diagnosis ICD-10	Indication for clozapine	Previous treatments	Treatment at time of clozapine initation	Treatment after clozapine except clozapine	Effect of clozapine	Clozapine: - dose - plasma levels^a^ - duration	Side effects
Case A Female 53 years	Profound MR (DF73), atypical autism (DF84.1) and organic mood affective disorder (DF06.3)	Aggressive- and self-injurious behaviour and affective symptoms	Olanzapine, melperone, quetiapine, clonazepam, chlorprothixene, carbamazepine, nitrazepam, promethazine, levomepromazine, haloperidol, periciazine, amitriptyline and ECT	Zuclopenthixol 70 mg and 10 mg PRN up to two times a day	Zuclopenthixol 10 mg PRN up to two times daily and melatonin 3 mg PRN at night	CGI-S 7→4. Reduced aggressive and self-injurious behaviour, affective symptoms and increased overall well-being	Clozapine 225 mg 120–165 ng/mL 2 years and 9 months	Mild constipation, which was managed with an osmotic laxative
Case B Male 47 years	Severe MR (DF72) and childhood autism (DF84)	Aggressive and self-injurious behaviour	Levomepromazine, carbamazepine, zuclopenthixol, alprazolam, pipamperone and lamotrigine	Olanzapine 30 mg and 5 mg PRN up to two times per day, zuclopenthixol 50 mg, alprazolam 4 mg and 1 mg PRN and lamotrigine 100 mg	Alprazolam 0.75 mg, lamotrigine 100 mg, zuclopenthixol 40 mg and zuclopenthixol 10 mg PRN up to once a day	CGI-S 6→4. Reduced aggressive and self-injurious behaviour. Increased overall well-being	Clozapine 450 mg 104–574 ng/mL 2 years	Mild constipation, which was managed with an osmotic laxative
Case C Female 68 years	Moderate MR (DF71), atypical autism (DF84.1) and encephalopathy (DG93.4)	Aggressive and self-injurious behaviour	Quetiapine, mirtazapine, venlafaxine, risperidone, zuclopenthixol, valproate, olanzapine, lamotrigine and ECT	Lamotrigine 200 mg, olanzapine 15 mg and oxazepam 15 mg PRN up to three times daily	—	CGI-S 5→5. No lasting effect on aggressive or self-injurious behaviour	Clozapine 250 mg 150–187 ng/mL 2 years	Mild constipation, which was managed with an osmotic laxative
Case D Male 31 years	Mild MR (DF70), pervasive developmental disorder, unspecified (D84.9) and persistent delusional disorder, NOS (DF22.9)	Aggressive behaviour and psychosis	Risperidone, zuclopenthixol, oxazepam and promethazine	Zuclopenthixol 50 mg	—	CGI-S 6→3.Reduced psychosis. No aggressive behaviour, and increased overall well-being	Clozapine 325 mg 112–564 ng/mL 5 years	Mild constipation, which was managed with an osmotic laxative Mild fatigue. Weight gain 21 kg over a period of 5 years. The patient was started on semaglutide and has so far lost 5 kg
Case E Male 56 years	Profound MR (DF73) and autistic disorder (DF84.0)	Aggressive behaviour and self-injurious behaviour	Risperidone, levomepromazine, periciazine, clonazepam and alprazolam	Valproate 1500 mg, zuclopenthixol 60 mg clonazepam 0.5 mg PRN	Valproate 900 mg, zuclopenthixol 20 mg and diazepam 3 mg	CGI-S 7→2. Reduced aggressive and self-injurious behaviour and increased overall well-being	Clozapine 225 mg 133–311 ng/mL 3 years	Mild hypersalivation and mild fatigue

Abbreviations: CGI-S, clinical global impression-severity of illness; ECT, electroconvulsive therapy; MR, mental retardation; PRN, pro re nata.

^a^Plasma levels reference range: 98–654 ng/mL.

## Data Availability

Data sharing is not applicable to this article as no datasets were generated or analysed during the current study.
